# Characterizing Venous Vasculatures of Hepatocellular Carcinoma Using a Multi-Breath-Hold Two-Dimensional Susceptibility Weighted Imaging

**DOI:** 10.1371/journal.pone.0065895

**Published:** 2013-06-14

**Authors:** Shi-Xin Chang, Guan-Wu Li, Yao Chen, Hong Bao, Lei Zhou, Jun Yuan, Dong-Mei Wu, Yong-Ming Dai

**Affiliations:** 1 Department of Radiology, Yueyang Hospital of Integrated Traditional Chinese & Western Medicine, Shanghai University of Traditional Chinese Medicine, Shanghai, China; 2 Shanghai Key Lab of Magnetic Resonance, East China Normal University, Shanghai, China; 3 Siemens Healthcare China, MR Collaboration NE Asia, Shanghai, China; Cornell University, United States of America

## Abstract

The aim of our study is to characterize the venous vasculatures of hepatocellular carcinoma (HCC) using a multi-breath-hold two-dimensional (2D) susceptibility weighted imaging (SWI) in comparison with conventional Magnetic Resonance Imaging (MRI) sequences. Twenty-nine patients with pathologically confirmed HCC underwent MR examination at a 3.0 T scanner. The number of venous vascularity in or around the lesion was counted and the image quality was subjectively evaluated by two experienced radiologists independently based on four image sets: 1) SWI, 2) T1-weighted sequence, 3) T2-weighted sequence, and 4) T1-weighted dynamic contrast-enhanced (DCE) sequence. Of the 29 patients, a total of 33 liver lesions were detected by both SWI and conventional MR sequences. In the evaluation of the conspicuity of venous vascularity, a mean of 10.7 tumor venous vessels per mass was detected by the SWI and 3.9 tumor vasculatures were detected by T1-weighted DCE (*P*<0.0001), while none was detected by T1-, T2-weighted sequences. The Pearson correlation coefficients between the lesion sizes and the number of tumor vasculatures detected by T1-weighted DCE was 0.708 (*P*<0.001), and 0.883 by SWI (*P*<0.001). Our data suggest that SWI appears to be a more sensitive tool compared to T1-weighted DCE sequence to characterize venous vasculature in liver lesions.

## Introduction

Hepatocellular carcinoma (HCC) is one of the five most common malignancies and the third leading cause of cancer-related deaths worldwide, with an increasing incidence due to hepatitis B and C viral infection [1]. In the process of HCC development, angiogenesis, as a concomitant yet indispensable process, will give rise to the growth of new vessels that provide the indispensible blood supply for tumor expanding beyond 1–2 mm^3^ [2]. So far, the changes in tumor micro-vessel density (MVD) or the measurement of the greatest dimension of all target lesions were adopted as the criterion to assess the treatment response of a tumor [3]–[4]. Nevertheless, the former totally overlooks the main issue that vasculature measurements done on biopsies or single slices from a lesion may be skewed by tumor heterogeneity and interior regions of necrosis, and the latter disregards tumor necrosis due to treatment, which is the objective of all effective loco-regional therapies widely used for HCC.

It is well known that blood vessel consists of artery, vein and capillary. An accurate evaluation of tumor vasculatures may provide a good estimation of pathological findings or help decide malignancy grades of hepatocellular carcinoma (HCC), which proposes a high demand for imaging methodology. Therefore, in vivo monitoring the abnormality of venous vasculatures may be of high clinical significance for deciding which treatment options in HCC to use, for example, selecting a surgical program and embolotherapy.

Magnetic Resonance Imaging (MRI), as a powerful invasive method, is desirable to measure vasculature for longitudinal studies of tumor development and treatment. Susceptibility Weighted Imaging (SWI) is well known as a three dimensional (3D) gradient echo sequence (GRE) based technique which employs phase information as an additional source of contrast to visualize the susceptibility changes induced by different substances such as blood products (hemosiderin and ferritin), deoxygenated blood, calcium, iron, and small vein depiction in various physiological and pathological conditions [5]–[9]. Recently, with the advent of a multi-breath-hold two dimensional (2D) GRE-sequence-based SWI, several abdominal applications, which improve detection of iron containing nodules of cirrhosis liver and better characterize hemorrhages in HCC, were well performed [10]–[11]. We hypothesized that 2D SWI would be consistently more sensitive to vascular structure in tumor than conventional MR imaging techniques, as 3D SWI did in neurology application [5], [9], [12]. However, to our knowledge, there was no related study carried out to date.

The purpose of this retrospective study was to assess whether the multi-breath-hold 2D SWI is a more powerful tool for the visualization and characterization of venous vasculatures in HCC than current-used T1-weighted DCE and conventional MR imaging techniques.

## Materials and Methods

### Ethics Statement

All research procedures were approved by the appropriate institutional research ethics committee (the Institutional Review Board of Yueyang Hospital of Integrated Traditional Chinese & Western Medicine, Shanghai University of Traditional Chinese Medicine) and were conducted in accordance with the Declaration of Helsinki. Written informed consent for each study was obtained independently for all patients.

### Participants

In this study a total of 29 consecutive patients (26 males and 3 females, 42 to 79 years old, mean age 60.1 years at the time of diagnosis) with a diagnosis of HCC were recruited from the Department of Oncology between January 1^st^, 2012 and September 30^st^, 2012 and had conducted a specialized MR examination. Criteria of patient enrollment were as follows: 1) all patients were in stable clinical conditions with no known contraindications against MRI; 2) ability to sustain at least a 20 second breath hold; 3) no patient had any surgical or local treatment of the lesion before the MR examination; 4) lacking of diagnosed or self-reported other malignancies and 5) no iron overloaded patients. Three of 29 patients had multifocal HCC lesions (two patients with 2 lesions per case, and one patient with 3 lesions) the remaining 26 patients with a solitary lesion. All tumors were pathologically proved by means of either surgical specimens (n = 16) or radiographic-guided biopsy (n = 13). The pathological diagnosis HCC was performed according to the standards described in the 2010 WHO Classification of Tumors of the Digestive System [13].

### MR Imaging

MR examination for all subjects was performed on a 3.0 Tesla whole body system (MAGNETOM Verio, Siemens Healthcare, Erlangen, Germany) using a standard 12-channel body matrix coil for acquisition. MRI examination was completed by the same certified-MRI technician.

The MR examination consisted of four MR sequences: 1) transverse respiratory-navigated 2D T2-weighted fat-suppressed turbo spin echo sequence (TSE, TR/TE 4000/78 msec; flip angle 140°; No. of slices, 30; slice thickness, 5 mm; intersection gap, 1 mm; matrix, 168×320; ETL, 9); 2) multi-breath-hold 2D T1-weighted fast low-angle shot sequence (FLASH, TR/TE 140/2.5 msec; flip angle, 70°; No. of slices, 30; slice thickness, 5 mm; intersection gap, 1 mm; matrix, 180×320); 3) multi-breath-hold 2D SWI (TR/TE 150/10 msec; flip angle, 20°; No. of slices, 30; slice thickness, 5 mm; intersection gap, 1 mm; matrix, 187×384; voxel size 1.5×1.0×5.0 mm^3^) and 4) T1-weighted DCE imaging with a 3D fat-suppressed FLASH (TR/TE 4/1.4 msec; flip angle, 9°; No. of slices, 64; slice thickness, 5 mm; no intersection gap; matrix, 180×320). The T1-weighted DCE was performed once before and three times after intravenous administration of gadopentetate dimeglumine (Magnevist; Bayer Healthcare, Berlin, Germany). Gadopentetate dimeglumine (standard dose, 0.1 mmol per kilogram of body weight) was administered at a rate of 2 ml/sec followed by a 20-ml saline flush using a power injector (Spectris; Medrad, Pittsburgh, PA). The acquisitions were performed at 25, 60, and 180 seconds after contrast agent administration during the hepatic arterial dominant phase, portal venous phase, and equilibrium phase, respectively.

For abdominal SWI, a multi-breath-hold 2D GRE sequence based SWI (Work-in-Progress sequence, WIP#608, Siemens Healthcare, Erlangen, Germany) was used. Parallel imaging was performed using generalized auto calibrating partially parallel acquisition (GRAPPA) with an acceleration factor of 2. A complete SWI scan consisted of 3 contiguous 10-slice transverse acquisitions through the liver, with duration of each acquisition suitable for a single breath-hold (16 sec). Therefore, 30 slices covering the entire liver could be acquired in less than 1 minute and 17 seconds: 16 seconds for each of three breath-holds, an instruction period of 3 seconds per breath-hold, and two breaks for 8 to 10 seconds between breath-holds 1, 2 and 2, 3. SWI post-processing was done inline and included the complex steps as previously described by Dai [14]. For all four sequences, only T1-weighted DCE was performed with intravenous injection and the field of view (FOV) was optimized to the patients’ body habitus, 280×210–280×380 mm^2^.

### Image Analysis

First of all, the T1-weighted DCE dataset was selected as a reference for analysis, as it has been extensively used for measurements associated with angiogenesis [14]–[16]. From the T1-weighted DCE dataset of every patient, a single representative slice of the collected data that covering the lesions was chosen, in consensus, by two experienced radiologists, on which most vasculatures were displayed. Then, corresponding slices that matched or most closely matched the representative slice were chosen from the T1-, T2-weighted and 2D SWI datasets by comparing the position of the liver. For each patient, the number of representative slices was decided by the number of lesions. Tumor vasculatures were enumerated in the representative slice of liver mass.

After creating the combined representative dataset for each patient, two radiologists reviewed all MR images on a commercially available imaging workstation (Syngo Multimodality Workplace [MMWP]; Siemens Healthcare). The two experienced radiologists were blinded to previous clinical MR interpretations, pathologic results, and sequences used. For 2D SWI, only low-signal structures that could be traced on contiguous images and appeared as long, linear or curved cylindrical tube-shaped structures with clear boundaries were considered to represent tumor veins [12]. For T1-weighted DCE sequence, the number of tumor veins in both portal venous phase and equilibrium phase was counted respectively. For each of the representative slices, the number of tumor vascularity was counted and the image quality (that is, the conspicuity of tumor vascularity) was subjectively evaluated by calculating the vessel-normal liver parenchyma contrast-to-noise ratio (CNR). The signal intensity (SI) of the vessels in tumor and normal liver parenchyma, as well as the standard deviation (SD) of background noise was measured by operator-defined regions of interest (ROIs). ROIs for vessels and liver parenchyma were drawn in the same location on images of each sequence. Background noise was measured using ROIs positioned immediately ventral to the right anterior abdominal wall. The vessel-normal liver parenchyma CNR was calculated using the following formula [11], [17]:
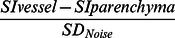
(1)where 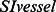 stands for the SI of vessel, 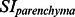 for the SI of normal liver parenchyma and 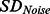 for the standard deviation of background noise.

If multiple tumor vasculatures were observed, the smallest one was served as the effective signal of tumor vascularity for its relatively lower SI, compared to larger ones, would strictly test the abilities of different imaging methods to visualize venous vasculatures. The SI of tumor venous vasculature and tumor, the SD of background noise, and lesion maximum diameter (centimeter) were measured twice and averaged. All differences in readers’ interpretation were resolved by a panel judgment including an additional board-certified abdomen-radiologist.

### Statistical Analysis

All data analysis was performed using the Statistical Package and Service Solutions in Windows, Version 17.0 (SPSS, Chicago, IL, USA). Shapiro-Wilk test was performed to test the normality distribution of the data. Intraclass correlation coefficient (ICC) was used to determine the levels of inter-observer variability in quantitative analysis of tumor vasculatures, which were defined by each reader based on images acquired with different MRI sequences (T1WI, T2WI, T1-weighted DCE and non-contrast-enhanced SWI). A paired *t* test was performed to compare whether there was any significant difference in the quantitative analysis of tumor abnormal vascularity detected among different sequences, with respect to the number of detected tumor vasculatures per lesion. Pearson’s correlation coefficients were used to show the relationships between lesion size and the number of tumor vascularity on different sequences. All statistical tests were two-tailed, and *P*<0.05 was considered statistically significant.

## Results

### Basic Characteristics of the Mass and Inter-reader Agreement

**Figure 1:**
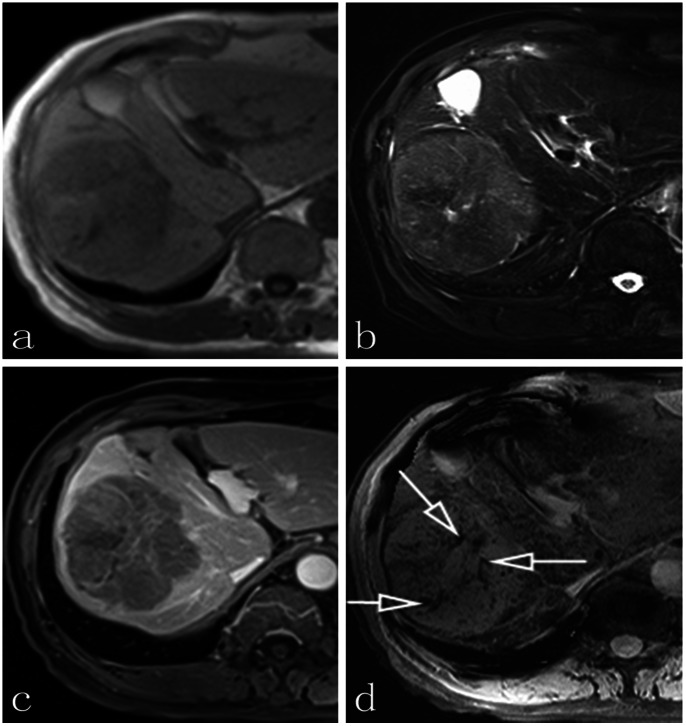
MR images of a 59-year-old man with a solitary HCC. a: no obvious tumor vasculature is visible in the axial T1-weighted imaging or b: the axial T2-weighted imaging; c: the contrast-enhanced axial T1-weighted image shows the mass with irregularly enhancement and no obvious tumor veins were detected; d: noncontrast-enhanced SWI shows considerably more detail of the internal architecture than T1-weighted DCE. Scattered linear hypointense signals (arrows) suggest radiating veins in the centre of the mass.

**Figure 2:**
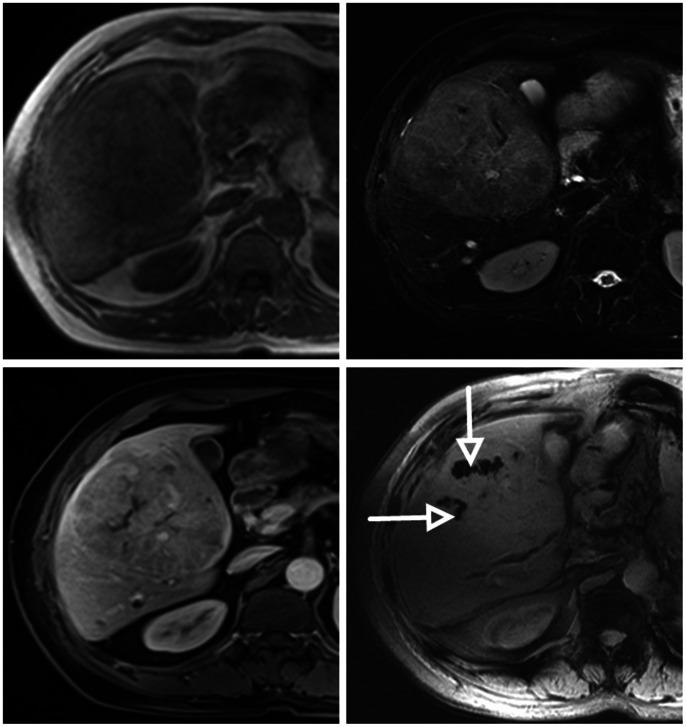
A case of a 72-year-old man with HCC. **a, b, and c correspond to T1 precontrast, the axial T2-weighted imaging, and T1-weighted DCE, respectively; d: axial non-contrast SWI indicates intratumorally linear vasculatures or curved cylindrical tube-shaped structures with clear boundaries (arrows).**

In the 29 HCC patients 33 liver lesions were detected by both SWI and conventional MRI sequences. Lesion sizes were ranged from 2.03 to 9.26 cm (median 4.51±1.83 cm). Tumor venous vessels cannot be revealed on conventional T1WI or T2WI, whereas the SWI sequence from 29 patients depicted tumor veins in or around the lesion (Figures 1 and 2). As shown in Table 1, in the quantitative analysis, inter-observer agreement for the number of tumor venous vasculature within HCC and the conspicuity of tumor vascularity was excellent for determining the number of tumor vascularity (ICC = 0.827 for the T1-weighted DCE sequence, *P*<0.001; 95% confidence interval, 0.613–0.922, and ICC = 0.970 for the SWI sequence, *P*<0.001; 95% confidence interval, 0.935–0.987) and for calculating the venous vessel-normal liver parenchyma contrast-to-noise ratio (ICC = 0.839 for the T1-weighted DCE sequence, *P*<0.001; 95% confidence interval, 0.697 to 0.924, and ICC = 0.929 for the SWI sequence, *P*<0.001; 95% confidence interval, 0.838 to 0.958).

**Table 1:** The levels of interobserver variability in quantitative analysis of tumor veins determined on both T1-weighted DCE and Non-contrast-enhanced SWI.

	ICC	95% confidence interval	*F* test with true value 0	
			value	*P*-values
Number of tumor veins on DCE-T1WI	0.827	0.613 to 0.922	5.764	<0.001
Number of tumor veins on SWI	0.970	0.935 to 0.987	66.180	<0.001
CNR of tumor veins on DCE-T1WI	0.839	0.697 to 0.924	12.972	<0.001
CNR of tumor veins on SWI	0.929	0.838 to 0.958	14.138	<0.001

CNR, contrast-to-noise ratio; DCE-T1WI, contrast-enhanced T1-weighted imaging; ICC, Intraclass correlation coefficient; SWI, susceptibility-weighted imaging.

### The Quantitative Analysis of Tumor Vasculatures

Mean (SD) number of tumor venous vessels of HCC seen on both two sequences observed by the two radiologists are displayed in Table 2. The CNR of tumor veins on SWI (36.7±11.3) was significantly higher than that on the T1-weighted DCE sequence (26.5±10.2) (*P*<0.001). The number of tumor veins on both portal venous phase and equilibrium phase was evaluated respectively; the number of tumor vasculatures per mass seen on the portal venous phase (1.7±0.9) was significantly lower than that on the equilibrium phase (3.9±1.8) (*P*<0.001). In the final result, therefore, we selected the equilibrium phase of the T1-weighted DCE dataset compared to SWI to depict tumor vascularity. Considering the number of tumor venous vessels of HCC, according to the first observer, there was a mean of 3.7 tumor vasculatures per mass seen on T1-weighted DCE (SD, 1.6; maximum, 7; minimum, 1) and 11.1 tumor vasculatures on SWI (SD; 6.0; maximum, 23; minimum, 2). For the second radiologist, a mean of 4.2 tumor vasculatures were found on T1-weighted DCE (SD, 1.9; maximum, 9; minimum, 1) compared with 10.4 tumor vasculatures on the SWI sequence (SD, 5.6; maximum, 21; minimum, 2). Two pictorial cases are shown in Figures 1 and 2.

**Table 2:** The number of tumor veins per HCC mass detected on both CE-T1 and SWI sequences for both observers.

	The first reviewer				The second reviewer				Average			
	SWI	DCE-T1WI	*t*	*P*-value	SWI	DCE-T1 WI	*t*	*P*-value	SWI	DCE-T1 WI	*t*	*P*-value
Number of tumor veins	11.1±6.0	3.7±1.6	7.586	<0.001	10.4±5.6	4.2±1.9	7.072	<0.001	10.7±5.7	3.9±1.8	7.670	<0.001
Contrast-to-noise ratio	34.7±10.9	25.9±10.5	4.051	<0.001	38.6±12.1	27.8±9.5	4.546	<0.001	36.7±11.3	26.5±10.2	4.318	<0.001

DCE-T1, contrast-enhanced T1-weighted imaging; SWI, susceptibility-weighted imaging.

Values are represented as mean ± SD.

*P*-values were calculated using paired *t*-tests between SWI and DCE-T1 sequence.

**Figure 3:**
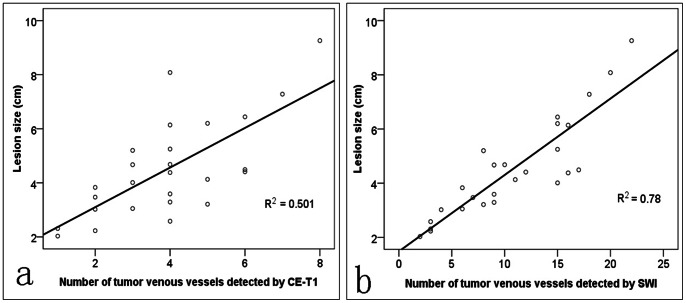
Correlations between the lesion maximum size and tumor venous vasculatures detected on contast-enhanced T1-weighted imaging (a) and on SWI (b) (***P*** **<0.001 for all).**

The Pearson’s correlation coefficients between the lesion sizes and the number of tumor vasculatures detected by T1-weighted DCE was 0.708 (*P*<0.001), and 0.883 by SWI (*P*<0.001) were shown in Figure 3.

## Discussion

SWI has become an important tool in the neuroradiology imaging in the past decade [12]. Assessment of tumor tissue vascularization and visualization of the dilated draining vein by using SWI in patients with brain tumors could be valuable to help grading intracranial gliomas [18]. Recently, SWI is also shown to be beneficial in quantification of tumor hemorrhage, and hence, correlate hemorrhage with tumor staging in abdominal organs of human body, such as liver [11], [19]. Mie et al. first implemented SWI on the kidney revealing that through variation of the phase mask even small susceptibility differences become visible [20]. A recent study by Dai et al. has demonstrated that abdominal SWI appears to provide the most sensitive information to detect siderotic nodules in cirrhotic liver using a 2D multi- breath-hold SWI technique [10]. These results represent initial experiences with SWI of the human abdomen. In the present study, we attempt to make this technology applicable in clinical abdominal MRI for characterizing and detecting tumor venous vascularity in HCC. We found the CNR between tumor veins and normal liver parenchyma on SWI was significantly higher than that of T1-weighted DCE sequence, indicating that tumor vascularity detected by SWI exhibited superior contrast. In other word, the tumor tissue vascularization appeared more hypointensive in comparison with the background tumor tissue.

The presence of arterial enhancement in conventional DCE-MRI of the liver has been used as a valuable tool for the detection of HCC in clinic practice. Current clinical practice guidelines suggest that HCC can be accurately diagnosed without biopsy in patients with the backgrounds of liver cirrhosis by a demonstration of hypervascularity in the arterial phase and early washout phase of T1-weighted DCE [21]. However, there are some hyperintensive lesions on unenhanced images in which it is difficult to discriminate [15]. Additionally, some hypovascular HCCs in which early arterial enhancement cannot be demonstrated on DCE-MRI or CT. Namely, the potential of detecting tumor vascularity on T1-weighted DCE appears limited. In this preliminary study, we reported, for the first time, the use of non-contrast enhanced SWI to guide the quantitative evaluation of tumor venous vasculatures of HCC. The main finding in the present study is that SWI is superior to the T1-weighted DCE sequence for detecting the tumor venous vasculatures of HCC while no tumor vessels were detected with conventional T1WI or T2WI, thus indicating the feasibility of liver SWI for HCC patients.

HCC is a hypervascular solid tumor, in which angiogenesis plays a crucial role. Arterialization and sinusoidal capillarization are two important vascular changes associated with tumor angiogenesis in HCC. The status of angiogenesis in HCC correlates with the disease progression and prognosis, and thus provides a target for novel therapeutic approaches [2], [22]. On the other hand, SWI is especially sensitive to venous vasculature, even to vessels smaller than a voxel [6]–[7]. Detecting abnormalities in venous vasculature inside tumors also provides critical information about the tumor’s blood supply [23]. Our findings confirmed non-contrast enhanced SWI is much better than conventional T1WI, T2WI, and T1-weighted DCE sequence for showing venous vasculatures in HCC, and the correlation between the lesion mean size and the number of tumor vasculatures detected by SWI (*r* = 0.883) is higher than that of T1-weighted DCE (*r* = 0.708). It has been shown that as the arterial vasculatures increase, the grade of histological differentiation in HCC decreases. Although no histopathological differentiation between veins and arteries is available at present, if we hypothesize that vascular hyperplasia in tumor occurs equivalently between arterial and venous portions, the contiguous low-signal regions (suggesting venous vessels) depicted on the SWI sequence may symbolize the equal number of arterial hyperplasia noted on histopathology [12].

A dynamic monitoring of vascular changes in tumor is of great significance in clinical practice. First, it is an extremely useful auxiliary means to monitor the response of the tumor to antiangiogenic therapy, which is a new and promising method for HCC therapy. MVD is considered to be the “golden” standard of reference for the proof of angiogenesis in tumor patients [3]. Nevertheless, MVD assessed with immunocytochemical techniques presents only a partial picture of tissue microvasculature, failing to reflect the functional and dynamic features of angiogenesis. Second, vascularity within a tumor can be spatially or temporally heterogeneous [24]. Data obtained from a small portion of the tumor mass may not be representative of the entire tumor response. As a result, assessment of angiogenesis in the liver tumor by traditional MVD poses special limitations. On the other hand, 3D dynamically monitoring of vascular changes in antiangiogenic therapy has put forward an urgent demand and expectation on imaging modalities of how they can be used to determine the efficacy of these treatments [2]. Recently, DCE-MRI has become a promising, attractive approach to evaluate tumor microvasculature in solid tumors [25]. Unlike the limited anatomic information of MVD, DCE-MRI studies describe a general picture of the functional status with information on tumor microenvironment. However, the longer acquisition time and complicated post-processing are unrealistic in busy clinical practice, especially 4D tissue DCE-MRI (measuring pharmacokinetic parameters in microvasculature by quantifying the transfer of a contrast agent from the vascular space to the extravascular and extracellular space over time.) [16]. Likewise, perfusion CT may be applied for quantification of tumor vascular density and angiogenesis, as well as for evaluation of tumor response to antiangiogenic agents [26]. However, the disadvantage of ionizing radiation limits its application. On the other hand, the risk of contrast agent injection should also be taken into consideration. It is safer to perform SWI without contrast agent. Park et al. had demonstrated that non-contrast enhanced SWI is sufficient to assist the grading of gliomas and SWI in combination with contrast agents do not appear to add additional values [18]. In general, non-contrast enhanced SWI is a noninvasive, nonradiative and feasible quantitative method in a single examination.

Nevertheless, several limitations of this study should be acknowledged. First, the research sample size of patients with HCC was relatively small in this study, which may limit the statistical power. A larger clinical trial with more patients is needed in order to establish the clinical usefulness of SWI at 3T for evaluation of HCC. Second, the pathological examination was not performed to verify the venous vasculature as shown on SWI in the 16 surgical patients. Additionally, MVD shown by immunohistochemical staining was not routinely performed in our patients; therefore, we could not perform a correlation analysis between the number of intratumoral veins and the histopathological results. Third, since SWI is well known to enlarge the appearance of the veins [6], a single representative slice covering lesion maximum diameter was chosen to count tumor venous vessels, which may leads to false positive in the neighboring slices, This problem could be potentially addressed by counting the veins with a 3D scanning in the future. Finally, using a multi-breath-hold two-dimensional scan mode, there is a temporal limitation for the spatial resolution of the SWI sequence, and it is unable to completely match the representative slices obtained from the T1- weighted, T2- weighted, T1- weighted DCE, and SWI datasets.

In summary, the present study suggests that levels of angiogenesis within HCC can be clearly manifested by non-contrast enhanced SWI evaluation, which is a more sensitive tool compared to T1-weighted DCE sequence for detecting tumor venous vasculatures compared with conventional liver MR sequences. The feasibility of SWI in discriminating tumor vasculature is an innovative concept that requires further confirmation with the accumulation of more cases. The authors believe that the capabilities of SWI would be proved useful in future attempts to characterize tumors, and the total number of sequences needed for evaluation may be reduced.
